# New Weekly

**Published:** 1855-02

**Authors:** 


*New Weekly.*—We have received the first number of the "Medical Counsellor, or Weekly Gazette of Medical and Physical Sciences," R. Hills, M.D., Editor and Proprietor. It is published at Columbus, Ohio. Its typography is neat, and so far as we may judge from the present number, it is not lacking in editorial ability. Giving the same matter in four weekly numbers that the Buffalo Journal does in one monthly, it does not differ at all in the amount of labor required, except that the mechanical parts must require more constant attention. Dr. Hill has undertaken a task which will weigh heavily upon him as he progresses, especially as he has already two able competitors in his own state. We wish the new enterprise success in proportion to its deserts—and may they be many. H.

